# The effect of systemic levels of TNF-alpha and complement pathway activity on outcomes of VEGF inhibition in neovascular AMD

**DOI:** 10.1038/s41433-021-01824-3

**Published:** 2021-11-08

**Authors:** Adnan H. Khan, Charles O. Pierce, Gabriella De Salvo, Helen Griffiths, Marie Nelson, Angela J. Cree, Geeta Menon, Andrew J. Lotery

**Affiliations:** 1 Division of Clinical Neurosciences, Clinical and Experimental Sciences, Faculty of Medicine, University of Southampton, Southampton, UK; 2 Southampton Eye Unit, University Hospital Southampton NHS Foundation Trust, Southampton, UK; 3 Department of Ophthalmology, Frimley Park Hospital, Frimley Health NHS Foundation Trust, Camberley, UK

**Keywords:** Macular degeneration, Prognosis

## Abstract

**Background/Objectives:**

Systemic levels of pro-inflammatory cytokines and activated complement components affect the risk and/or progression of neovascular age-related macular degeneration (AMD). This study investigated the effect of serum pro-inflammatory cytokine levels and complement pathway activity on the clinical response to vascular endothelial growth factor (VEGF) inhibition in neovascular AMD.

**Methods:**

Sixty-five patients with a new diagnosis of neovascular AMD were observed over a six-month period in a single-centre, longitudinal cohort study. At each visit, the visual acuity score (VAS), central macular thickness (CMT), serum levels of CRP, pro-inflammatory cytokines (TNF-α, IL-1β, IL-2, IL-6 and IL-8), and complement pathway activity were measured. Participant DNA samples were sequenced for six complement pathway single nucleotide polymorphisms (SNPs) associated with AMD.

**Results:**

A statistically significant difference in VAS was observed for serum levels of TNF-α only: there was a gain in VAS (from baseline) of 1.37 for participants below the 1st quartile of mean concentration compared to a reduction of 2.71 for those above the 3rd quartile. Statistical significance was maintained after Bonferroni correction (*P* value set at <0.006). No significant differences in CMT were observed. In addition, statistically significant differences, maintained after Bonferroni correction, were observed in serum complement activity for participants with the following SNPs: *CFH* region (rs1061170), *SERPING1* (rs2511989) and *CFB* (rs641153). Serum complement pathway components did not significantly affect VAS.

**Conclusions:**

Lower serum TNF**-**α levels were associated with an increase in visual acuity after anti-VEGF therapy. This suggests that targeting pro-inflammatory cytokines may augment treatment for neovascular AMD.

## Introduction

Age-related macular degeneration (AMD), a progressive retinal disease that results in the loss of central vision, is predicted to affect 288 million people worldwide by 2040 [1]. Neovascular AMD (nAMD) is a result of choroidal neovascularisation (CNV) and leads to rapid vision loss. The mainstay of current treatment is inhibition of vascular endothelial growth factor (VEGF) [2]. The evidence base for a genetic component in AMD is significant, and numerous single nucleotide polymorphisms (SNPs) have been associated with a patient’s risk of developing AMD [3]. SNPs in genes of the complement pathway, including the complement factor B (*CFB*) gene region [4, 5], the *C2* [4, 5] and *C3* [6] genes have been reported to affect the risk of developing AMD.

Uncontrolled activation of the complement pathway is limited by a set of complement regulatory proteins: Factor H and Factor I (encoded by the *CFH* and *CFI* genes, respectively), regulate the alternative complement pathway [7], whereas the C1 inhibitor is a regulator of the classical pathway [8]. Genetic variants at the Regulators of Complement Activation (RCA) locus on *chromosome* 1, which contains the *CFH* gene, contributes to AMD risk [9–11], in addition to the *CFI* gene region on *chromosome* 4 [12–14], and the *SERPING1* gene that encodes the C1 inhibitor [15, 16].

Studies have shown elevated levels of complement activation fragments to be independently associated with AMD [17–19]. Furthermore, complement activation has been demonstrated to be associated with stage of AMD [20]. In addition, systemic activation of the alternative complement pathway and complement components is associated with AMD genotypes [21], including the *CFH* SNP rs1061170 (Y402H) [19] and the *CFI* region SNP rs10033900 [17, 21]. A meta-analysis by Hong et al. reported that treatment-naïve patients carrying the *CFH* SNP, rs1061170 (Y402H), were more likely to achieve an improved outcome to anti-VEGF treatment [22]. Furthermore, visual outcome was improved after anti-VEGF treatment for patients carrying a low-risk *CFH* genotype and low *CFH* risk score [23].

Expression of acute phase proteins and pro-inflammatory cytokines can also affect the risk of AMD development and/or progression: CRP is an acute phase protein and marker of systemic inflammation that is an independent risk factor for AMD [24]. IL-6 is a known cytokine stimulus of CRP release by the liver [25], and both have been associated with AMD progression [26]. CRP has been demonstrated to induce IL-8 expression by human retinal pigment epithelium (RPE) cell lines [27], and both IL-6 and IL-8 are expressed by RPE cells on complement activation [28], by degenerating RPE cells [29], and are associated with drusen formation [30]. Systemic levels of IL-6 have been found to be associated with the progression rate of geographic atrophy secondary to AMD [31]. In addition, patients with AMD have been shown to express higher levels of circulating IL-1β than age-matched controls [32]. IL-2 has been implicated in the pathogenesis of AMD as activation of IL-2 signalling pathways has been observed [33] and IL-2 contributes to extracellular matrix formation and the development of fibrosis in AMD [34]. TNF-α, a pro-inflammatory cytokine that is known to mediate CNV formation in experimental models by upregulating VEGF expression by RPE cells [35], has also been demonstrated to promote the angiogenic drive of active CNV lesions [36]. Patients with elevated levels of serum TNF-α have been shown to respond favourably to VEGF inhibition [32].

Although the studies mentioned above have investigated the role of complement pathway SNPs, complement pathway activity and systemic concentrations of pro-inflammatory cytokines on AMD pathogenesis, relatively few studies have investigated their functional effect on outcomes of VEGF inhibition. The primary aim of this study was to investigate the effect of serum levels of pro-inflammatory cytokines (TNF-α, IL-1β, IL-2, IL-6 or IL-8) and complement pathway activity on the clinical response to VEGF inhibition in neovascular AMD. A secondary aim was to investigate the effect of complement pathway SNPs, associated with AMD, on serum complement activity in the same cohort of patients.

## Materials and methods

### Study approval, registration and regulation

This study was conducted in accordance with the Research Governance Framework for Health and Social Care (2005) and Good Clinical Practice. Ethical approval was obtained from the National Research Ethics Committee (NRES) South Central- Southampton A. This study adhered to the tenets of the Declaration of Helsinki. The University Hospital Southampton NHS Foundation Trust was the sponsor of this study, and The University of Southampton undertook the research study. All patient samples and data were anonymised for the purpose of this study. Patient DNA and serum samples were stored for future studies. Procedures for handling, processing and storage of patient data were in compliance with the UK Data Protection Act (1998).

### Patient recruitment, consent, and investigation

Patients were recruited to the study after informed consent by the ophthalmology department of University Hospital Southampton NHS Foundation Trust. Patients were invited to take part if they met the principle inclusion criteria for the study: (1) over the age of 50; (2) a new diagnosis of neovascular AMD in one eye, treated with an initial loading dose of three, monthly Ranibizumab intravitreal injections; (3) White ethnicity (to limit any effects of ethnic variation on outcomes of VEGF inhibition in neovascular AMD). The exclusion criteria were: (1) bilateral diagnosis of neovascular AMD (one of the exploratory endpoints of the study was the development of nAMD in the second eye); (2) a macular co-pathology; (3) poor venous access that prevents a peripheral blood samples being taken.

**Fig. 1:**
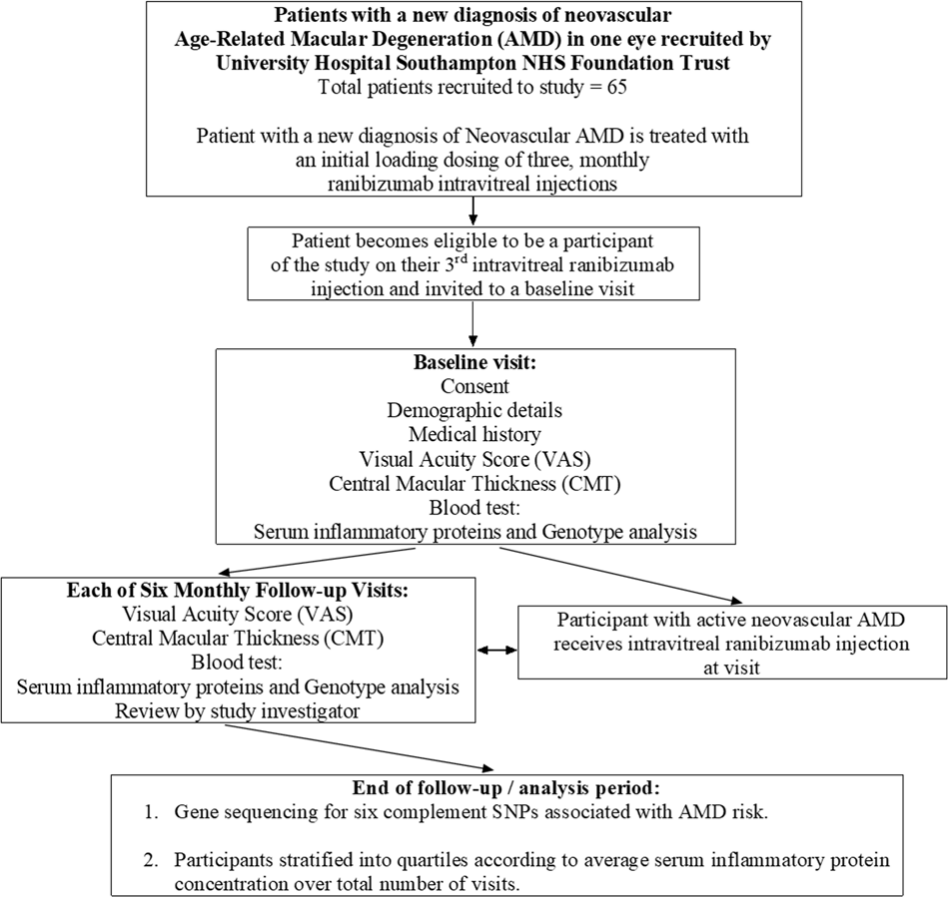
A flowchart diagram summarising the investigation pathway of 65 study participants with a new diagnosis of neovascular age-related macular degeneration (AMD). Peripheral blood samples were taken at seven visits (baseline visit and six follow-up visits), for serum and genotypic analysis.

All patients recruited to this study had a diagnosis of neovascular AMD, confirmed on fundus fluorescein angiography, that was made by a consultant ophthalmologist specialising in medical retina diseases. Indocyanine green angiography was carried out for patients to rule out polypoidal choroidal vasculopathy (PCV)- patients with PCV were not invited to take part in the study. Patients were eligible to enrol for the study after their third intravitreal Ranibizumab injection and subsequently invited to a baseline visit (Fig. 1). Informed consent was taken from participants at this visit, and their demographic details, medical history and baseline LogMAR visual acuity score (VAS) was recorded (number of letters on an ETDRS chart). A baseline central macular thickness (CMT) was also measured using optical coherence tomography (OCT) (Topcon, Berkshire, UK). A blood sample was taken at the baseline visit for serum cytokine and genetic analysis. Participants were reviewed by a study investigator and received treatment with an intravitreal ranibizumab injection if they had active neovascular AMD. Following the baseline visit, participants attended for six, monthly follow-up visits. At each visit, the VAS and CMT was recorded, a blood sample was taken, and the patient reviewed by a study investigator before any treatment for active disease.

### Detection of serum cytokine levels and activated end components of complement pathways

Serum was isolated from participant blood samples using standard density-gradient ultracentrifugation at 1355 × *g* for 10 min at 21 °C (Eppendorf, Stevenage, UK). Patient serum cytokine levels were measured using semi-quantitative assays by Meso Scale Discovery (Rockville, Maryland, USA) as per the manufacturer’s instructions. All cytokine measurements were undertaken in triplicate using the assay, and cytokine measurements were within the reading range of the kit. Functional assessment of classical and alternative pathway complement activity in patient serum samples was undertaken using Wieslab semi-quantitative ELISA Assays (SVAR Life Sciences, Malmo, Sweden) as per the manufacturer’s instructions. Measurement of activated end components of classical and alternative complement pathways was expressed as a percentage relative to the fluorescence intensity of the positive control, derived from human serum components, supplied with the testing kit.

### Genetic analysis

DNA was extracted from peripheral blood mononuclear cells of patient blood samples using erythrocyte lysis buffer (Fisher Scientific, Loughborough, UK) as previously described [37]. DNA concentrations were measured using the Nanodrop ND1000 spectrophotometer (Thermo Scientific, Wilmington, DE, USA). Sequence analysis of participant DNA samples was undertaken by LGC Genomics (Hoddesdon, UK) on the following six SNPs associated with the complement pathway and AMD risk: *CFH* region: rs1061170; *CFI* region: rs10033900; *SERPING1*/*C1-INH*: rs2511989; *CFB*: rs641153; *C2*: rs9332739; *C3*: rs2230199.

### Statistical analyses

The GraphPad Prism software version 8.2 (GraphPad Software, Lo Jolla, Ca, USA) was used for statistical analyses and graphical representation of the data obtained in this study. Assessment of normality of continuous variables was determined by quantile–quantile plots of the residuals using GraphPad Prism. The unpaired *t* test with Welch’s correction was used to determine statistically significant differences in changes of visual acuity scores, central macular thickness and percentage activity of activated end components of complement pathways compared to positive controls. Statistical significance was set at the *P* < 0.05 value. As this is a preliminary/pilot study, the patient sample size was determined using a rationale laid out by S.A. Julious where a sample size of at least 12 is recommended [38]. Our patient cohort was stratified into quartiles of ~16 in line with this recommendation.

## Results

### Serum classical or alternative complement pathway activity and functional response to anti-VEGF intravitreal injections

**Fig. 2:**
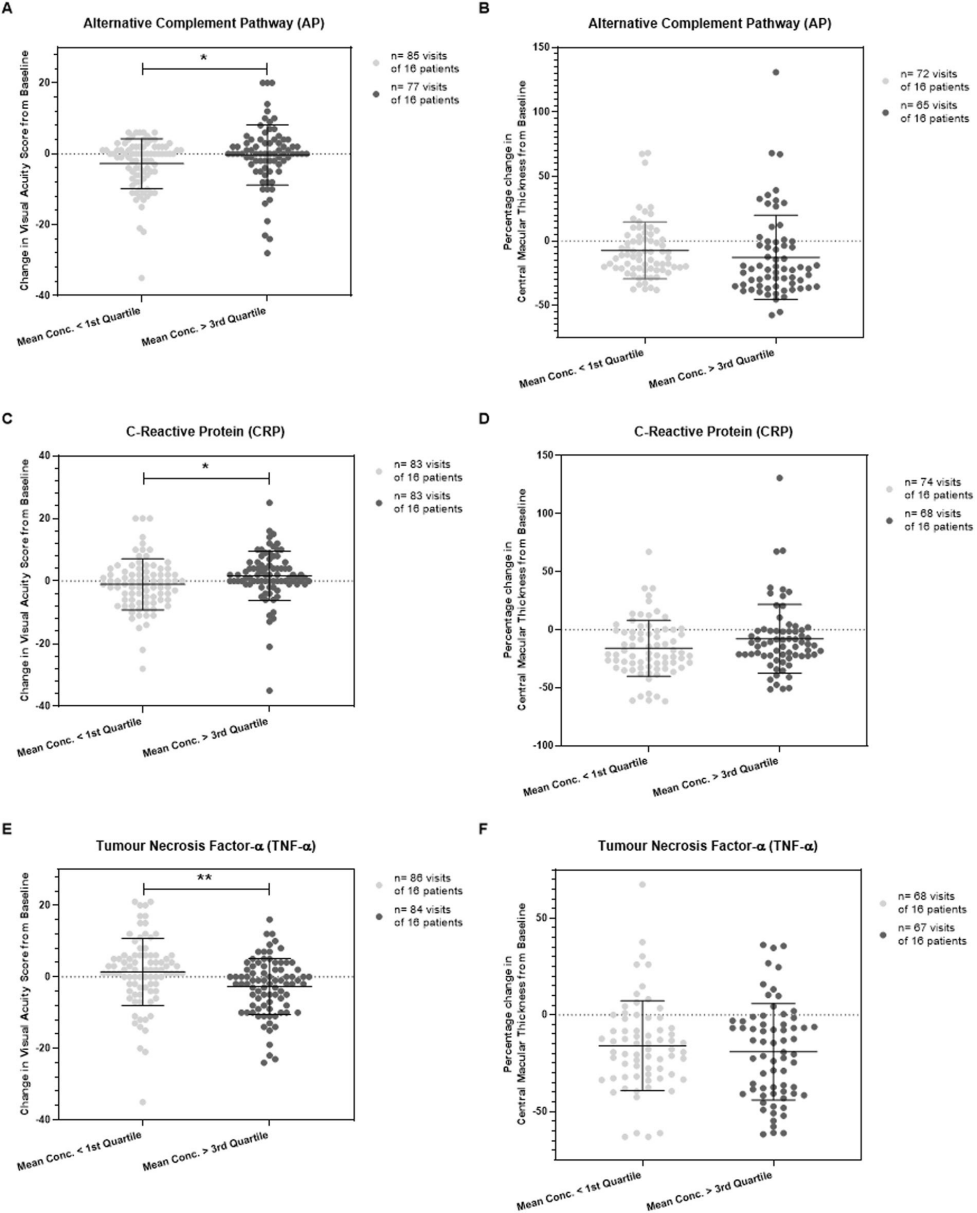
Change in visual acuity score (VAS) and central macular thickness (CMT) associated with serum concentration of alternative complement pathway components and inflammatory proteins. Study participants were stratified into quartiles according to average serum concentration of an investigated inflammatory protein (including pro-inflammatory cytokine) or complement pathway-specific components over seven study visits. The change in VAS from baseline at each visit is plotted for all study patients who had a mean serum concentration of inflammatory protein or complement pathway component below the first quartile and above the third quartile. The percentage change in CMT from baseline at each visit is also plotted for the same study participants. Shown in parts (**A**,**B**) are the results for alternative complement pathway components and change in VAS or CMT from baseline at each study visit for patients below or above the indicated quartiles; *P = 0.048. Shown in parts (**C**,**D**) are the results for C-Reactive Protein (CRP) and change in VAS or CMT from baseline at each study visit for patients below or above the indicated quartiles; *P = 0.029. Shown in parts (**E**,**F**) are the results for Tumour Necrosis Factor-α (TNF-α) and change in VAS or CMT from baseline at each study visit. **P = 0.0024. The unpaired *t* test, two-tailed, with Welch’s correction, was used to determine whether there was a statistically significant difference in VA or CMT change from baseline between groups.

A total of 65 patients with a new diagnosis of neovascular AMD were recruited to participate in this study (Fig. 1). Participant demographics are summarised in Table 1. Study participants were stratified into quartiles according to average serum concentration of an inflammatory protein over seven study visits, in order to amplify the functional effects of small changes in serum concentration (Table 1). The study first investigated any significant differences in the visual acuity score (VAS) or central macular thickness (CMT) change from baseline at each visit between participants who had a mean serum concentration of classical pathway (Supplementary Fig. 1A, B) or alternative pathway (Fig. 2A, B) complement components below the first quartile and above the third quartile. There was a statistically significant difference in the VAS change from baseline, −2.78 (SD = 7.01) vs. −0.34 (SD = 8.51) for mean serum alternative pathway components (*P* = 0.048), using an unpaired *t* test with Welch’s correction (Fig. 2A), but significance was not maintained after a Bonferroni correction was applied (*P* value set at <0.006).

**Table 1:** Study participant demographics and summary table.

Demographics						
Number of patients recruited to study			65			
Patient sex			48% (*n* = 31) male: 52% (*n* = 34) female			
Patient age (mean, SD)			79.7 (SD = 8.6)			
Smoking Status			Current Smoker: 11% (*n* = 7)			
			Ex-Smoker: 46% (*n* = 30)			
			Non-smoker: 43% (*n* = 28)			
Body Mass Index (BMI) (mean, SD)			26.8 (SD = 3.94)			
Co-morbidities			Hypertension: 47.7% (*n* = 31)			
			Asthma or COPD: 13.8% (*n* = 9)			
			Hyperlipidaemia: 13.8% (*n* = 9)			
			Diabetes (Type I or II): 12.3% (*n* = 8)			
			Nil co-morbidities declared: 16.9% (*n* = 11)			
Mean Ranibizumab intravitreal injections over six, monthly follow-ups (mean, SD)			1.94 (SD = 0.98)			
**Serum Inflammatory Protein; Units**			**Serum Concentration (Cohort): 1st** **Quartile; Median; 3rd** **Quartile; Variance**			
C-Reactive Protein (CRP)			1.23 mg/L; 2.81 mg/L; 7.82 mg/L; 571.21 mg/L			
Tumour Necrosis Factor-α (TNF-α)			0.098 pg/ml; 0.136 pg/ml; 0.179 pg/ml; 0.078 pg/ml			
Interleukin-1β (IL-1β)			0.004 pg/ml; 0.010 pg/ml; 0.023 pg/ml; 0.012 pg/ml			
Interleukin-2 (IL-2)			0.009 pg/ml; 0.017 pg/ml; 0.033 pg/ml; 0.010 pg/ml			
Interleukin-6 (IL-6)			0.180 pg/ml; 0.300 pg/ml; 0.492 pg/ml; 2.723 pg/ml			
Interleukin-8 (IL-8)			0.964 pg/ml; 1.462 pg/ml; 2.067 pg/ml; 61.87 pg/ml			
Complement Pathway Activity*			**Percentage activity relative to positive control (Cohort) 1st** **Quartile; Median; 3rd** **Quartile**			
Classical Complement Pathway (CP)			95.57%; 102.2%; 117.1%;			
Alternative Complement Pathway (AP)			89.29%; 97.27%; 115.7%;			
**Gene sequencing analysis undertaken on patient cohort**						
**Gene/DNA region**	**Reference SNP**	**Chromosome and Position (bp)**	**Major/Minor Allele**	**MAF**	**OR**	**Reference**
*CFH* region	rs1061170	Chr 1; 196,659,237	T/C	0.61	2.41	11
*CFI* region	rs10033900	Chr 4; 110,659,067	C/T	0.52	1.18	11
*SERPING1/C1-INH*	rs2511989	Chr 11; 57,134,901	G/A	0.45	0.63	15
*CFB*	rs641153	Chr 6; 31,914,180	C/T	0.05	0.54	11
*C2*	rs9332739	Chr 6; 31,903,804	G/C	0.02	0.46	11
*C3*	rs2230199	Chr 19; 6,718,387	G/C	0.24	1.53	11

*SD* standard deviation, *SNP* single nucleotide polymorphism, *MAF* minor allele frequency, *OR* odds ratio of AMD.

*Measurement of classical or alternative complement pathway activity is expressed as a percentage relative to the fluorescence intensity of a positive control.

### Serum inflammatory protein concentration and functional response to anti-VEGF intravitreal injections

Study participants were also stratified into quartiles according to mean serum concentration of CRP or a pro-inflammatory cytokine (TNF-α, IL-1β, IL-2, IL-6 or IL-8) over the seven study visits. Statistically significant differences initially observed for VAS change (Fig. 2C) for mean CRP concentration were not maintained after Bonferroni correction (*P* value was set at <0.006), and there were no significant differences in CMT change (Fig. 2D) using the unpaired *t* test with Welch’s correction.

Of the pro-inflammatory cytokines assessed, there was a statistically significant difference observed in the VAS change from baseline, 1.37 (SD = 9.40) vs. −2.71 (SD = 7.79), between participants for mean serum TNF-α concentration below the first quartile and above the third quartile, respectively (*P* = 0.0024), Fig. 2E. Significance was maintained after a Bonferroni correction was applied (*P* value was set at <0.006). No significant difference was observed in CMT change in these participants (Fig. 2F). In addition, no significant differences were observed in the VAS or CMT change from baseline between participants for mean serum concentration of IL-1β (Supplementary Fig. 1C, D), IL-2 (Supplementary Fig. 1E, F), IL-6 (Supplementary Fig. 1G, H), or IL-8 (Supplementary Fig. 1I, J).

### Complement pathway SNPs and activated complement end components

**Fig. 3:**
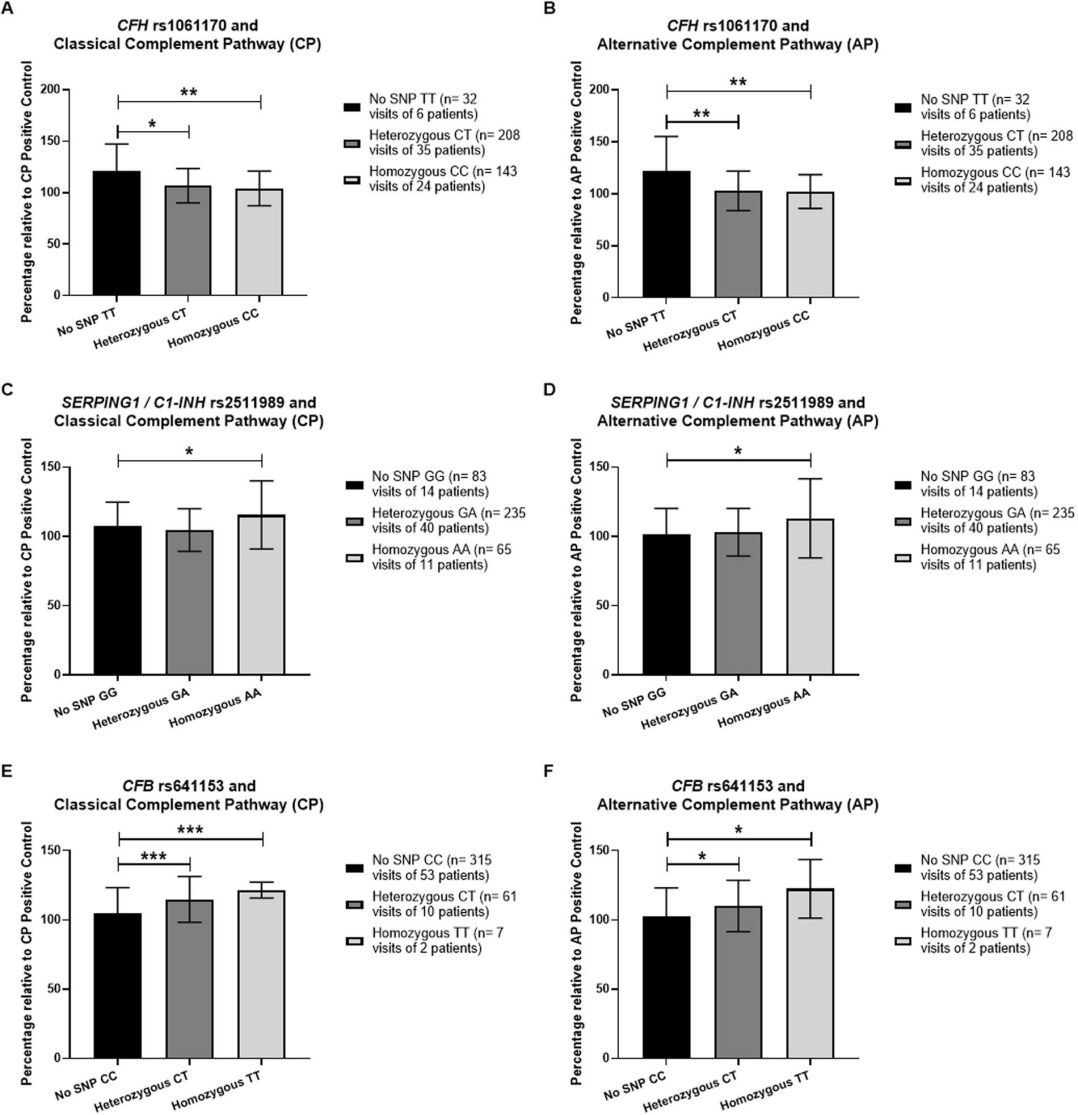
Classical or alternative complement pathway activity associated with single nucleotide polymorphisms (SNPs) in study participants. Study participants underwent DNA sequencing for the detection of six SNPs associated with the complement pathway and AMD risk. Assessment of serum levels of classical or alternative pathway complement components was undertaken on the same participants. The bar graphs (**A**–**F**) show the measurement of classical or alternative pathway complement activity on participants who express no SNP, are heterozygous, or homozygous for the following SNPs: *CFH* region: rs1061170 (**A**, **B**). *SERPING1*/*C1-INH*: rs2511989 (**C**, **D**). *CFB*: rs641153 (**E**, **F**). Measurement of activated end components specific for the classical or alternative complement pathway in serum samples is expressed as a percentage relative to the activity of the positive control. The unpaired *t* test, two-tailed, with Welch’s correction, was used to determine whether there was a statistically significant difference in classical or alternative pathway components between groups who had no SNP, were heterozygous for the indicated SNP, or homozygous for the indicated SNP. *P < 0.05; **P < 0.005; ***P < 0.0001.

All 65 study participants underwent DNA sequencing for the detection of six complement pathway SNPs reported to affect AMD risk. Measurement of classical or alternative pathway complement activity in the serum was undertaken at each study visit. Complement activity was expressed as a percentage relative to the positive control (based on human serum components) of the assay and could thus exceed 100%. For the *CFH* SNP rs1061170, a statistically significant reduction was observed: (1) in mean classical pathway complement activity in homozygous participants (16.7% reduction; *n* = 24; *P* = 0.0016) (Fig. 3A); (2) in mean alternative pathway activity in both homozygous (20.1% reduction; *n* = 24; *P* = 0.0019) and heterozygous participants (19.4% reduction; *n* = 35; *P* = 0.0025), Fig. 3B. For the *CFI* region SNP rs10033900, there was a statistically significant increase of 7.7%, after Bonferroni correction, in mean classical pathway activity, in both homozygous (*n* = 16; *P* = 0.0037) and heterozygous (n = 32; *P* = 0.0002) participants, Supplementary Fig. 2A, B. For the *SERPING1* / *C1-INH* SNP rs2511989, there was a significant increase in only the mean alternative pathway complement activity of 11.8% in homozygous participants (*n* = 11; *P* = 0.005), maintained after Bonferroni correction Fig. 3C, D.

A significant increase in mean classical pathway complement activity of 16.3% was observed, after Bonferroni correction (*P* value set at <0.008), in patients who were homozygous for the *CFB* SNP rs641153 (*n* = 2; *P* < 0.0001) and 9.7% who were heterozygous (*n* = 10; *P* < 0.0001), Fig. 3E. Similarly, a significant increase in alternative complement pathway activity of 7.3% was observed, after Bonferroni correction, in heterozygous patients (*n* = 10; *P* = 0.0069), Fig. 3F. No differences were observed for the *C2* SNP rs9332739 or *C3* SNP rs2230199 (Supplementary Fig. 2C–F).

## Discussion

The primary aim of this study was to investigate the effect of serum pro-inflammatory cytokine levels and complement pathway activity on the clinical response to VEGF inhibition in neovascular AMD. After Bonferroni correction, a statistically significant difference was observed only in VAS (change from baseline) between participants stratified into quartiles by mean TNF-α serum concentration [a gain of 1.37 for participants below the 1st quartile compared to a reduction of 2.71 above the 3rd quartile]. This was not associated with significant changes in CMT. This study supports both pre-clinical and clinic findings showing a small, but significant overall impact of systemic levels of TNF-α on CNV lesions and clinical responses to VEGF inhibition. In a previous study using a murine model of laser-induced CNV, inhibition of TNF-α with intraperitoneal injections of infliximab or etanercept led to significantly reduced CNV lesion size and pathological fluorescein leakage [39]. Furthermore, in a non-controlled trial, infusions of the anti-TNF-α chimeric monoclonal antibody, infliximab, in neovascular AMD demonstrated non-progression of the disease in almost half of the treated patients and regression of exudative lesions without significant fibrous scarring [40]. There was, however, no placebo group in this trial. Other small studies using intravitreal anti-TNF-α therapy combined with bevacizumab showed beneficial effects [41].

This study also investigated the effect of complement pathway SNPs, associated with AMD, on serum classical or alternative pathway complement activity in the same cohort of patients. A statistically significant, but modest, reduction (after Bonferroni correction) of classical pathway activity was observed in participants who were homozygous for the *CFH* region SNP rs1061170, in addition to a reduction in alternative pathway activity in participants who were either homozygous or heterozygous for this SNP. Furthermore, participants who were either homozygous or heterozygous for the *CFI* region SNP rs10033900 had a statistically significant increase in classical pathway activity, despite Factor I being better recognised as a regulator of the alternative pathway [7]. Despite these differences, this study demonstrated no significant differences in VAS or CMT change from baseline between participants below the 1st quartile and above the 3rd quartile of mean serum classical or alternative pathway complement components. Therefore, although statistically-significant, modest, differences in serum complement activity were observed in participants with *CFI*, *CFH* and other complement pathway SNPs tested, this did not translate to real-world, significant differences in visual response to anti-VEGF treatment.

A recent study by Heesterbeek et al. demonstrated higher systemic levels of activated complement in patients with intermediate AMD (who demonstrated the highest serum complement activation), geographic atrophy and inactive neovascular AMD compared to patients with active nAMD [20]. This raises the question of whether significant increases in complement activity were not observed in this study as all patients had active nAMD. Interestingly, it was demonstrated in a study by Keir et al., that anti-VEGF intravitreal injections in neovascular AMD patients resulted in increased levels of complement components (C3a, C4a and C5a) in the aqueous humour [42], and this was elevated in patients with earlier relapses of active nAMD compared to those with later relapses.

This study focused on measuring overall complement pathway activation (via the activated end products of complement activation) rather than a specific activated complement component, e.g. C3d. Commercially-available Wieslab assays (Svar Life Sciences) were used in this study, which are optimised to detect activation of the complement pathway using human serum. This assay was previously used to demonstrate a significant elevation in the activity of the alternative complement pathway in AMD patients with genetic variants in *CFB* and *C3* compared to controls [19]. An alternative method to detect elevated systemic complement activation in our study could have been to calculate the C3d/C3 ratio from the plasma concentration of these complement components. This method was also used (in addition to the Wieslab assays) in recent studies to detect systemic complement activation in AMD patients with genetic variants [19, 20]. It will be interesting to see whether there is any difference between the Wieslab assay method and C3d/C3 ratio to measure systemic complement activation in our cohort of participants.

This study analysed individual genetic variants and their effect on complement pathway activation, which demonstrated some statistically-significant effects. Previous studies have demonstrated that the association of gene variants with complement activation in AMD patients may be stronger when undertaking haplotype analysis [43] compared to single variant analysis. An additive effect of complement pathway risk SNPs has been suggested to lead to an additive risk of disease [44]. In the recent study by Heesterbeek et al. the association of AMD stage with complement activation was greatest in patients with haplotypes that were associated with the highest levels of complement activation [20]. It will be interesting to undertake haplotype analysis to investigate the effect of overall complotype on the outcomes of VEGF inhibition in AMD in our patient cohort.

Studies have suggested that AMD pathogenesis is driven primarily by dysregulation of immune mediators locally within the eyes rather than circulating levels of these mediators. A study by Agawa et al. demonstrated that intravitreal anti-VEGF treatment (with bevacizumab) itself significantly raised intraocular levels of IL-6 and IL-8 [45], both implicated in AMD pathogenesis. A subsequent study, in contrast, demonstrated a reduced intraocular concentration of IL-6 after intravitreal aflibercept injection [46]. Peripheral blood mononuclear cells (PBMCs), particularly monocytes, from AMD patients have been demonstrated to produce higher levels of IL-8 than age-matched controls [47], and it has been speculated that these cells could migrate to the macula to secrete additional IL-8.

The concept of AMD being a disease of systemic or local complement dysregulation was previously discussed by our group nearly eight years ago [48]. Studies have suggested that SNPs associated with complement activation increase AMD risk by a combination of systemic activation of complement and dysregulation of complement activation in local tissues [49]. It is unknown whether altered, systemic levels of complement in AMD are the result of AMD-associated gene variants whose effects are expressed in all tissues, or the result of circulating levels of complement that reach the choroid and retina to contribute to AMD pathogenesis. It is also thought that AMD pathogenesis is driven by a combination of locally-expressed complement factors [50, 51] in addition to systemic complement proteins which lead to local effects in tissues, e.g. the FH-related proteins (FHR) such as FHR-4 [52]. This study did not demonstrate any statistically-significant functional effects, after Bonferroni correction, of elevated or reduced systemic complement pathway activity on outcomes of VEGF inhibition.

There were several limitations to this study, the most significant being the small cohort size of 65 participants. The primary reason for this is the challenge in recruiting a large number of patients for a study in which blood tests are taken every visit over seven months, in addition to an intravitreal injection where required. Fortunately, no participants dropped out of this study and all participant data from each visit was used in the analysis. Another limitation to this study was taking blood tests (for serum cytokine and complement pathway activity) after the loading dose of three, monthly intravitreal ranibizumab injections. The ethical regulations of this study meant that the priority was for patients to receive their loading dose of intravitreal anti-VEGF injections prior to enrolment in the study and for blood tests to be undertaken subsequently. Although the biggest gains in visual activity usually take place during the loading phase of intravitreal anti-VEGF injections, measurement of visual outcomes took place in this study from the starting point of all participants having received their three, monthly intravitreal injections. Although there is emerging evidence of intravitreal ranibizumab injections affecting serum concentrations of pro-inflammatory cytokines (including a transient reduction of serum TNF-α levels) in patients with diabetic macular oedema [53], similar/significant evidence has not been demonstrated in the context of nAMD. Similarly, increased serum levels of TNF-α have been demonstrated in treatment-naïve patients with diabetic macular oedema [54], but not in nAMD patients [32].

In light of serum TNF-α levels being associated in this study with small, but significant effects on visual acuity after treatment with anti-VEGF intravitreal injections, it would be worth investigating this cytokine in larger cohorts to determine if this effect can be replicated. This would determine if systemic levels of TNF-α could be used to identify non-responders to anti-VEGF treatment.

### Summary

#### What was known before

- Systemic levels of pro-inflammatory cytokines affect the risk and/or progression of neovascular AMD.
- Elevated levels of complement components and SNPs in the complement pathway are associated with AMD risk.

#### What this study adds

- Systemic levels of TNF-alpha could be used to identify non-responders to anti-VEGF treatment in neovascular AMD.
- Although AMD risk SNPs in the complement pathway affected serum complement activity, there was no direct effect of serum complement levels on outcomes of VEGF inhibition in this study.

## Supplementary information

Supplementary Figure 1

Supplementary Figure 2

## References

[1] Wong WL, Su X, Li X, Cheung CM, Klein R, Cheng CY (2014). Global prevalence of age-related macular degeneration and disease burden projection for 2020 and 2040: a systematic review and meta-analysis. Lancet Glob Health.

[2] Khandhadia S, Cherry J, Lotery AJ (2012). Age-related macular degeneration. Adv Exp Med Biol.

[3] Fritsche LG, Igl W, Bailey JN, Grassmann F, Sengupta S, Bragg-Gresham JL (2016). A large genome-wide association study of age-related macular degeneration highlights contributions of rare and common variants. Nat Genet.

[4] Gold B, Merriam JE, Zernant J, Hancox LS, Taiber AJ, Gehrs K (2006). Variation in factor B (BF) and complement component 2 (C2) genes is associated with age-related macular degeneration. Nat Genet.

[5] Maller J, George S, Purcell S, Fagerness J, Altshuler D, Daly MJ (2006). Common variation in three genes, including a noncoding variant in CFH, strongly influences risk of age-related macular degeneration. Nat Genet.

[6] Maller JB, Fagerness JA, Reynolds RC, Neale BM, Daly MJ, Seddon JM (2007). Variation in complement factor 3 is associated with risk of age-related macular degeneration. Nat Genet.

[7] Lachmann PJ (2019). The story of complement factor I. Immunobiology.

[8] Davis AE, Whitehead AS, Harrison RA, Dauphinais A, Bruns GA, Cicardi M (1986). Human inhibitor of the first component of complement, C1: characterization of cDNA clones and localization of the gene to chromosome 11. Proc Natl Acad Sci USA.

[9] Edwards AO, Ritter R, Abel KJ, Manning A, Panhuysen C, Farrer LA (2005). Complement factor H polymorphism and age-related macular degeneration. Science.

[10] Haines JL, Hauser MA, Schmidt S, Scott WK, Olson LM, Gallins P (2005). Complement factor H variant increases the risk of age-related macular degeneration. Science.

[11] Yu Y, Bhangale TR, Fagerness J, Ripke S, Thorleifsson G, Tan PL (2011). Common variants near FRK/COL10A1 and VEGFA are associated with advanced age-related macular degeneration. Hum Mol Genet.

[12] Fagerness JA, Maller JB, Neale BM, Reynolds RC, Daly MJ, Seddon JM (2009). Variation near complement factor I is associated with risk of advanced AMD. Eur J Hum Genet.

[13] Alexander P, Gibson J, Cree AJ, Ennis S, Lotery AJ (2014). Complement factor I and age-related macular degeneration. Mol Vis.

[14] Ennis S, Gibson J, Cree AJ, Collins A, Lotery AJ (2010). Support for the involvement of complement factor I in age-related macular degeneration. Eur J Hum Genet.

[15] Ennis S, Jomary C, Mullins R, Cree A, Chen X, Macleod A (2008). Association between the SERPING1 gene and age-related macular degeneration: a two-stage case-control study. Lancet.

[16] Lee AY, Kulkarni M, Fang AM, Edelstein S, Osborn MP, Brantley MA (2010). The effect of genetic variants in SERPING1 on the risk of neovascular age-related macular degeneration. Br J Ophthalmol.

[17] Reynolds R, Hartnett ME, Atkinson JP, Giclas PC, Rosner B, Seddon JM (2009). Plasma complement components and activation fragments: associations with age-related macular degeneration genotypes and phenotypes. Invest Ophthalmol Vis Sci.

[18] Sivaprasad S, Adewoyin T, Bailey TA, Dandekar SS, Jenkins S, Webster AR (2007). Estimation of systemic complement C3 activity in age-related macular degeneration. Arch Ophthalmol.

[19] Smailhodzic D, Klaver CC, Klevering BJ, Boon CJ, Groenewoud JM, Kirchhof B (2012). Risk alleles in CFH and ARMS2 are independently associated with systemic complement activation in age-related macular degeneration. Ophthalmology.

[20] Heesterbeek TJ, Lechanteur YTE, Lores-Motta L, Schick T, Daha MR, Altay L (2020). Complement activation levels are related to disease stage in AMD. Invest Ophthalmol Vis Sci.

[21] Scholl HP, Charbel Issa P, Walier M, Janzer S, Pollok-Kopp B, Borncke F (2008). Systemic complement activation in age-related macular degeneration. PLoS ONE.

[22] Hong N, Shen Y, Yu CY, Wang SQ, Tong JP (2016). Association of the polymorphism Y402H in the CFH gene with response to anti-VEGF treatment in age-related macular degeneration: a systematic review and meta-analysis. Acta Ophthalmol.

[23] Shah AR, Williams S, Baumal CR, Rosner B, Duker JS, Seddon JM (2016). Predictors of response to intravitreal anti-vascular endothelial growth factor treatment of age-related macular degeneration. Am J Ophthalmol.

[24] Seddon JM, Gensler G, Milton RC, Klein ML, Rifai N (2004). Association between C-reactive protein and age-related macular degeneration. JAMA.

[25] Gabay C, Kushner I (1999). Acute-phase proteins and other systemic responses to inflammation. N. Engl J Med.

[26] Seddon JM, George S, Rosner B, Rifai N (2005). Progression of age-related macular degeneration: prospective assessment of C-reactive protein, interleukin 6, and other cardiovascular biomarkers. Arch Ophthalmol.

[27] Wang Y, Bian ZM, Yu WZ, Yan Z, Chen WC, Li XX (2010). Induction of interleukin-8 gene expression and protein secretion by C-reactive protein in ARPE-19 cells. Exp Eye Res.

[28] Lueck K, Busch M, Moss SE, Greenwood J, Kasper M, Lommatzsch A (2015). Complement stimulates retinal pigment epithelial cells to undergo pro-inflammatory changes. Ophthalmic Res.

[29] Higgins GT, Wang JH, Dockery P, Cleary PE, Redmond HP (2003). Induction of angiogenic cytokine expression in cultured RPE by ingestion of oxidized photoreceptor outer segments. Invest Ophthalmol Vis Sci.

[30] Hageman GS, Luthert PJ, Victor Chong NH, Johnson LV, Anderson DH, Mullins RF (2001). An integrated hypothesis that considers drusen as biomarkers of immune-mediated processes at the RPE-Bruch’s membrane interface in aging and age-related macular degeneration. Prog Retin Eye Res.

[31] Krogh Nielsen M, Subhi Y, Molbech CR, Falk MK, Nissen MH, Sorensen TL (2019). Systemic levels of interleukin-6 correlate with progression rate of geographic atrophy secondary to age-related macular degeneration. Invest Ophthalmol Vis Sci.

[32] Nassar K, Grisanti S, Elfar E, Luke J, Luke M, Grisanti S (2015). Serum cytokines as biomarkers for age-related macular degeneration. Graefes Arch Clin Exp Ophthalmol.

[33] Makarev E, Cantor C, Zhavoronkov A, Buzdin A, Aliper A, Csoka AB (2014). Pathway activation profiling reveals new insights into age-related macular degeneration and provides avenues for therapeutic interventions. Aging (Albany NY).

[34] Jing R, Qi T, Wen C, Yue J, Wang G, Pei C (2019). Interleukin-2 induces extracellular matrix synthesis and TGF-beta2 expression in retinal pigment epithelial cells. Dev Growth Differ.

[35] Wang H, Han X, Wittchen ES, Hartnett ME (2016). TNF-alpha mediates choroidal neovascularization by upregulating VEGF expression in RPE through ROS-dependent beta-catenin activation. Mol Vis.

[36] Xu J, Zhu D, He S, Spee C, Ryan SJ, Hinton DR (2011). Transcriptional regulation of bone morphogenetic protein 4 by tumor necrosis factor and its relationship with age-related macular degeneration. FASEB J.

[37] Miller SA, Dykes DD, Polesky HF (1988). A simple salting out procedure for extracting DNA from human nucleated cells. Nucleic Acids Res.

[38] Julious SA (2005). Sample size of 12 per group rule of thumb for a pilot study. Pharm Stat.

[39] Shi X, Semkova I, Muther PS, Dell S, Kociok N, Joussen AM (2006). Inhibition of TNF-alpha reduces laser-induced choroidal neovascularization. Exp Eye Res.

[40] van Hagen PM, Baarsma GS, van Bilsen CE, Kuijpers RW, van Laar JA, van der Ent M (2014). A noncontrolled trial of anti-TNF-alpha chimeric monoclonal antibody (infliximab, Remicade((R))) in exudative age-related macular degeneration. Acta Ophthalmol.

[41] Freitas LG, Isaac DL, Tannure WT, Gabriel LA, Reis RG, Rassi AR (2013). Intravitreal bevacizumab combined with infliximab in the treatment of choroidal neovascularization secondary to age-related macular degeneration: case report series. Arq Bras Oftalmol.

[42] Keir LS, Firth R, Aponik L, Feitelberg D, Sakimoto S, Aguilar E (2017). VEGF regulates local inhibitory complement proteins in the eye and kidney. J Clin Invest.

[43] Lores-Motta L, Paun CC, Corominas J, Pauper M, Geerlings MJ, Altay L (2018). Genome-Wide Association Study reveals variants in CFH and CFHR4 associated with systemic complement activation: implications in age-related macular degeneration. Ophthalmology.

[44] Harris CL, Heurich M, Rodriguez de Cordoba S, Morgan BP (2012). The complotype: dictating risk for inflammation and infection. Trends Immunol.

[45] Agawa T, Usui Y, Wakabayashi Y, Okunuki Y, Juan M, Umazume K (2014). Profile of intraocular immune mediators in patients with age-related macular degeneration and the effect of intravitreal bevacizumab injection. Retina.

[46] Sato T, Takeuchi M, Karasawa Y, Enoki T, Ito M (2018). Intraocular inflammatory cytokines in patients with neovascular age-related macular degeneration before and after initiation of intravitreal injection of anti-VEGF inhibitor. Sci Rep.

[47] Lechner J, Chen M, Hogg RE, Toth L, Silvestri G, Chakravarthy U (2017). Peripheral blood mononuclear cells from neovascular age-related macular degeneration patients produce higher levels of chemokines CCL2 (MCP-1) and CXCL8 (IL-8). J Neuroinflammation.

[48] Warwick A, Khandhadia S, Ennis S, Lotery A (2014). Age-related macular degeneration: a disease of systemic or local complement dysregulation?. J Clin Med.

[49] Hecker LA, Edwards AO, Ryu E, Tosakulwong N, Baratz KH, Brown WL (2010). Genetic control of the alternative pathway of complement in humans and age-related macular degeneration. Hum Mol Genet.

[50] Loyet KM, Deforge LE, Katschke KJ, Diehl L, Graham RR, Pao L (2012). Activation of the alternative complement pathway in vitreous is controlled by genetics in age-related macular degeneration. Invest Ophthalmol Vis Sci.

[51] Schick T, Steinhauer M, Aslanidis A, Altay L, Karlstetter M, Langmann T (2017). Local complement activation in aqueous humor in patients with age-related macular degeneration. Eye (Lond).

[52] Cipriani V, Lores-Motta L, He F, Fathalla D, Tilakaratna V, McHarg S (2020). Increased circulating levels of Factor H-Related Protein 4 are strongly associated with age-related macular degeneration. Nat Commun.

[53] Gnanasekaran S, Bandala-Sanchez E, Kolic M, Churilov L, Rogers SL, McAuley AK (2020). The association between intravitreal ranibizumab therapy and serum cytokine concentrations in patients with diabetic macular edema. Mol Vis.

[54] Kocabora MS, Telli ME, Fazil K, Erdur SK, Ozsutcu M, Cekic O (2016). Serum and aqueous concentrations of inflammatory markers in diabetic macular edema. Ocul Immunol Inflamm.

