# Supplementary material for: The effect of systemic levels of TNF-alpha and complement pathway activity on outcomes of VEGF inhibition in neovascular AMD
Source: Eye (Lond). 2021 Nov 8;36(11):2192–9. doi: 10.1038/s41433-021-01824-3 (PMC9581945; doi:10.1038/s41433-021-01824-3)
Supplement: Supplementary file 1 — Supplementary Figure 1 [file 41433_2021_1824_MOESM1_ESM.docx]

**Supplementary Figure 1. Change in visual acuity score (VAS) and central macular thickness (CMT) associated with serum concentration of classical complement pathway components and inflammatory proteins.** Study participants were stratified into quartiles according to average serum concentration of an investigated inflammatory protein (including proinflammatory cytokine) or complement pathway-specific components over seven study visits. The change in visual acuity score (VAS) from baseline at each visit is plotted for all study patients who had a mean serum concentration of inflammatory protein or complement pathway component below the first quartile and above the third quartile. The percentage change in central macular thickness (CMT) from baseline at each visit is also plotted for the same study participants. Shown in parts **A-B** are the results for classical complement pathway components and change in VAS or CMT from baseline at each study visit for patients below or above the indicated quartiles. Shown in parts **C-J** are the results for the indicated cytokines and change in VAS or CMT from baseline at each study visit for patients below or above the indicated quartiles. The unpaired *t* test, two-tailed, with Welch’s correction, was used to determine whether there was a statistically significant difference in VA or CMT change from baseline between groups. No results were statistically significant.
