# Supplementary material for: The effect of systemic levels of TNF-alpha and complement pathway activity on outcomes of VEGF inhibition in neovascular AMD
Source: Eye (Lond). 2021 Nov 8;36(11):2192–9. doi: 10.1038/s41433-021-01824-3 (PMC9581945; doi:10.1038/s41433-021-01824-3)
Supplement: Supplementary file 2 — Supplementary Figure 2 [file 41433_2021_1824_MOESM2_ESM.docx]

**Supplementary Figure 2. Complement pathway activity associated with single nucleotide polymorphisms (SNPs) in study participants.** Study participants underwent DNA sequencing for the detection of six single nucleotide polymorphisms (SNPs) associated with the complement pathway and AMD risk. Assessment of serum levels of classical or alternative pathway complement components was undertaken on the same participants. The bar graphs **A-F** show the measurement of classical or alternative pathway complement activity on participants who express no SNP, are heterozygous, or homozygous for the following SNPs: *CFI* region: rs10033900 (**A-B**). *C2*: rs9332739 (**C-D**). *C3*: rs2230199 (**E-F**). Measurement of activated end components specific for the classical or alternative complement pathways in serum samples is expressed as a percentage relative to the activity (fluorescence intensity) of the positive control supplied with the testing kit. The unpaired *t* test, two-tailed, with Welch’s correction, was used to determine whether there was a statistically significant difference in classical or alternative pathway components between groups who had no SNP, were heterozygous for the indicated SNP, or homozygous for the indicated SNP. ***P*<0.005.
